# Relationship of genetic causes and inhibin B in non obstructive azoospermia spermatogenic failure

**DOI:** 10.1186/s12881-017-0456-x

**Published:** 2017-09-06

**Authors:** Qing-jun Chu, Rui Hua, Chen Luo, Qing-jie Chen, Biao Wu, Song Quan, Yong-tong Zhu

**Affiliations:** Department of Obstetrics and Gynecology, Center for Reproductive Medicine, Nanfang Hospital/ The First School of Clinical Medicine, Southern Medical University, Guangzhou, 510515 China

**Keywords:** Non obstruction azoospermia, Karyotype, Y chromosome microdeletion, Inhibin B

## Abstract

**Background:**

Chromosomal disorders in non obstructive azoospermia (NOA) may have an important influence on spermatogenesis, which may be reflected by the serum inhibin B levels. Till now, few studies have concerned the relationship of genetic causes and inhibin B in NOA.

**Methods:**

In this retrospective study, 322 men with NOA in Center for Reproductive Medicine, Nanfang Hospital, Southern Medical University were collected. The level of follicle stimulating hormone (FSH), inhibin B, Y chromosome microdeletion test (YCMD) and karyotype were measured.

**Results:**

Abnormal karyotypes were present in 38.5% of NOA, and YCMD were present in 18.0%, there was a high correlation between karyotypes and YCMD (χ^2^ = 11.892, *P* < 0.001). The level of inhibin B in chromosomal abnormality from lowest to highest was 46,XX (or 45,X), 47, XXY, mosaics, polymorphisms, inversion and translocation. And the level of inhibin B within Non-AZF a&b region deletion was higher than AZF a&b microdeletion.

**Conclusion:**

According to the level of inhibin B, spermatogenesis in chromosomal abnormality from lowest to highest was 46,XX (or 45,X), 47, XXY, mosaics, polymorphisms, inversion and translocation. And spermatogenesis within Non-AZF a&b region deletion was better than AZF a&b microdeletion.

## Background

Azoospermia is a situation defined by the World Health Organization that absent sperm in the ejaculate semen sample even after an extended centrifugation [1]. The popularity of azoospermia is around 1% in the male population, and about 10%–15% in infertile men. Non obstructive azoospermia (NOA) is one of the most challenging subset. Some NOA patients are suitable using intracytoplasmic sperm injection (ICSI) as infertility treatment because their wives are difficult to obtain pregnancy naturally or by drug treatment. ICSI could help them to be biological parents by utilizing their single spermatozoa which have been obtained from testicular sperm aspiration (TESA), testicular sperm extraction (TESE) or micro-TESE.

Meanwhile, the technology of ICSI may carry the risk of passing on chromosomal disorders to their offspring. Karyotype analysis is recommended when ICSI needs to be carried out or sperm concentration < 5 × 10^6^ /ml [2]. The correlation between a higher frequency of chromosomal abnormality and the severity of the testicular phenotype became a consensus gradually, and genetic examine became a part of the procedure selected in diagnostic workup.

So far, the studies about genetic causes in NOA were focused on Klinefelter syndrome (KS) which has been systematically researched from hormone concentrations to testicular function [3, 4]. On the other hands, the studies in parts of genetic causes such as chromosome polymorphism, translocation or inversion disorders were relatively fewer in number [5]. Therefore, it will be interesting to consider the genetic causes in NOA, which maybe help us to understand the different spermatogenesis influenced by genetic causes.

The serum inhibin B levels were dramatically lower in male with a spermatogenic defect. Inhibin B, which is likely to be the crucial feedback regulator of FSH secretion [6], is associated well with FSH in sperm concentration, and thus support them as serum characters of spermatogenesis [7]. Therefore, the influences of chromosomal disorders in NOA on spermatogenesis may be reflected by the serum inhibin B levels. This study was designed for the first time to focus on relationship of genetic causes and inhibin B in NOA, summary the data and respond this question.

## Methods

### Study design

From 2012 to 2014, a total of 322 men with NOA were included in study. All patients were infertile men who referred to Center for Reproductive Medicine, Nanfang Hospital, Guangzhou, China. This study was approved by the Clinical Medical Local Ethical Review Committee of Southern Medical University. Informed written consent was obtained from every participant.

Both criteria of NOA and obstructive azoospermia (OA) were no sperm in semen after an extended centrifugation, and differentiation between NOA and OA was distinguished by history, physical examination, laboratory investigation, chromosomal karyotype, Y chromosome microdeletion (YCMD) test and testis biopsy [8]. NOA was classified according to histopathological findings as hypospermatogenesis by a decreased number of spermatozoa, as Sertoli cell only (SCO) by absent germ cells, and as maturation arrest (MA) by germ cells with arrest and without spermatozoa. Parts of NOA did not perform biopsy as the volume of testis only 1–4 ml.

### Hormone analysis

Blood samples were collected and centrifuged after clotting, then stored at −20 °C until analysis. Serum inhibin B concentrations were measured using an availably purchased, double antibody enzyme-linked immuno sorbent assay kits (Serotec, Oxford, UK). The detection limit was 20 pg/mL with coefficient variation of 12%–17%.

### Genetic analysis

Chromosome analysis was detected using G-banding. Peripheral blood samples were collected, lymphocytes were cultured in RPMI 1640 (eBioscience, San Diego, California), phytohaemagglutinin (Sigma-Aldrich, St. Louis, Missouri) and fetal bovine serum (Thermo Scientific HyClone, Logan, Utah), then treated with colcemid. More than 20 G-banding of metaphase chromosomes were detected for each patient. Chromosomal disorders were described according to the International System for Human Cytogenetic Nomenclature [9].

Peripheral blood samples were collected, genomic DNA was extracted using DNA isolation kit (Applied Biosciences, Carlsbad, New Mexico) in according with the manufacturer’s protocol. After the extraction of DNA, Y chromosome loci AZFa, AZFb, AZFc and AZFd were amplified by polymerase chain reaction with specific primers. The sequence of primers is shown in Table 1.

**Table 1:** Primers of selected genes

Gene name	Primers (forward/reverse)
SY133	5-ATTTCTCTGCCCTTCACCAG-3 5-TGATGATTGCCTAAAGGGAA-3
SY146	5-ACAAAAATGTGGCTCAGGGA-3 5-AAATAGTGTGCCCACCCAAA-3
SY153	5-GCATCCTCATTTTATGTCCA-3 5-CAACCCAAAAGCACTGAGTA-3
SY155	5-ATTTTGCCTTGCATTGCTAG-3 5-TTTTTAAGCCTGTGACCTGG-3
SY157	5-CTTAGGAAAAAGTGAAGCCG-3 5-CCTGCTGTCAGCAAGATACA-3
SY158	5-CTCAGAAGTCCTCCTAATAGTTCC-3 5-ACAGTGGTTTGTAGCGGGTA-3
SY182	5-TCAGAAGTGAAACCCTGTATG-3 5-GCATGTGACTCAAAGTATAAGC-3
SY238	5-AACAAGTGAGTTCCACAGGG-3 5-GCAAAGCAGCATTCAAAACA-3
SY254	5- GGGTGTTACCAGAAGGCAAA −3 5-GAACCGTATCTACCAAAGCAGC-3
SY255	5-GTTACAGGATTCGGCGTGAT-3 5-CTCGTCATGTGCAGCCAC-3
SY272	5-GGTGAGTCAAATTAGTCAATGTCC-3 5-CCTTACCACAGGACAGAGGG-3
SY277	5-GGGTTTTGCCTGCATACGTAATTA-3 5-CCTAAAAGCAATTCTAAACCTCCAG-3
SY283	5-CAGTGATACACTCGGACTTGTGTA-3 5-GTTATTTGAAAAGCTACACGGG-3

### Statistical analysis

Calculations were analyzed by using SPSS 19.0 software (SPSS Inc., Chicago, Illinois, USA). All numeric data were presented as the mean value ± standard deviation. Frequencies were expressed as percentages. The statistical analysis was performed Students t-test between 2 groups, whereas χ^2^ -test was used for comparison of proportions. Comparison of mean values among more than three groups was performed by using analysis of variance test. The correlation between karyotypes and Y chromosome microdeletions was calculated by chi-squared-test. Differences between the values were considered statistically significant when *P* < 0.05.

## Results

### Characteristics of patients

**Fig. 1:**
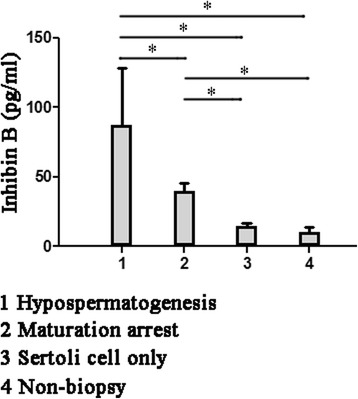
Serum inhibin B levels in different histopathological patients. Values represent the mean ± standard error. Significance between groups is indicated by *p < 0.05

There were a total of 322 men with NOA from 2012 to 2014. Mean age was 29.83 ± 4.54 years. Mean testis volume was 5.8 ± 5.4 ml. Mean serum FSH was 18.02 ± 7.16 mIU/ml, mean serum free testosterone was 6.04 ± 3.06 pg/ml and mean serum inhibin B was 46.01 ± 43.17 pg/ml. Compared to NOA with biopsy, the testis volume was smaller, serum FSH was higher, serum free testosterone was lower in NOA with non- biopsy. Compared to men with hypospermatogenesis, those with SCO, MA and non-biopsy had decreased inhibin B on univariate analysis (Fig. 1, *p* < 0.05); Compared to men with MA, those with SCO and non-biopsy had decreased inhibin B on univariate analysis (Fig. 1, *p* < 0.05). The incidence of genetic abnormality was 43.5% in overall men. Abnormal karyotypes were present in 38.5% of NOA, and YCMD were present in 18.0% (Table 2).

**Table 2:** Patient histopathological characteristics

	Biopsy			Non-biopsy	Overall	
	Hypospermatogenesis	Maturation arrest	Sertoli cell only			
No	169	64	45	44	322	
Age (y)	27.78 ± 3.26	27.40 ± 3.29	30.33 ± 4.05	29.67 ± 4.09	29.83 ± 4.54	0.65(a)
Testis volumn (ml)	12.4 ± 4.3	10.7 ± 4.0	8.0 ± 1.4	1.5 ± 1.6	5.8 ± 5.4	<0.05(a)
FSH (mIU/ml)	8.01 ± 3.57	14.74 ± 3.57	34.02 ± 7.02	35.65 ± 6.61	18.02 ± 7.16	<0.05(a)
Free testosterone (pg/ml)	9.81 ± 1.73	8.69 ± 2.68	5.62 ± 1.73	2.55 ± 2.03	6.04 ± 3.06	<0.05(a)
Inhibin B (pg/ml)	86.96 ± 40.97	39.72 ± 5.91	14.73 ± 2.23	10.71 ± 3.23	46.01 ± 43.17	<0.05(a)
Abnormal karyotypes (%)	26(8.0)	23(7.1)	21(6.5)	42(13.0)	112(34.8)	<0.05(b)
YCMD	13(4.0)	12(3.7)	15(4.7)	18(5.6)	58(18.0)	<0.05(b)

*YCMD* Y chromosome microdeletion

a:1-way ANOVA; b: chi-square test

The statistical data presented in Table 3 show a high correlation (χ^2^ = 11.892, continuity corrected *P* < 0.001) between karyotypes and Y chromosome microdeletions.

**Table 3:** Correlation between karyotypes and Y chromosome microdeletions

		karyotypes		
		abnormal	normal	Total
YCMD	microdeletions	32	26	58
	No deletion	80	184	264
	Total	112	210	322

χ^2^ = 11.892, continuity corrected *P* < 0.001

*YCMD* Y chromosome microdeletion

### Abnormal karyotypes and inhibin B

**Fig. 2:**
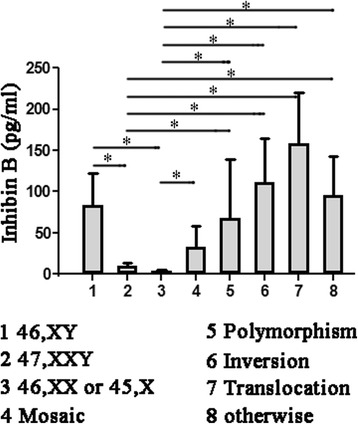
Serum inhibin B levels in different karyotypes patients. Values represent the mean ± standard error. Significance between groups is indicated by *p < 0.05

As shown in Table 4, normal karyotype (46, XY) was detected in 210 (65.2%) of the 322 NOA patients examined. The most common type of chromosomal abnormality was 47, XXY, with an incidence of 10.6%, and the serum inhibin B level was 10.49 ± 3.81 pg/ml. The serum inhibin B level in other sex chromosomal abnormalities (46,XX or 45,X) was even lower, 3.74 ± 1.43 pg/ml. But inhibin B level in mosaics of these karyotypes, such as 46,XY/47,XXY, 46,XY/45,X, was elevated to 33.23 ± 25.68 pg/ml. Compared to men with other subtypes, those with 47, XXY, 46,XX or 45,X had decreased inhibin B on univariate analysis (Fig. 2, *p* < 0.05).

**Table 4:** Abnormal karyotypes and inhibin B

	No	%	Inhibin B (pg/ml)
46,XY	210	65.2	84.00 ± 38.16
47,XXY	34	10.6	10.49 ± 3.81
46,XX or 45,X	8	2.5	3.74 ± 1.43
Mosaic karyotypes	12	3.7	33.23 ± 25.68
Polymorphism of sexual chromosome	27	8.4	68.58 ± 70.37
Chromosomal inversion	10	3.1	111.14 ± 53.24
Chromosomal translocation	8	2.5	158.01 ± 62.34
Abnormal karyotypes, otherwise	13	4.0	96.43 ± 46.31
Total	322	100.0	84.01 ± 63.17
		*P* Value	<0.05 (1-way ANOVA)

The second common type was polymorphism of sexual chromosome, such as 46,XY,1qh+,46,X,Yqh+, with an incidence of 8.8%, and inhibin B concentration was 68.58 ± 70.37 pg/ml. Inhibin B was high in chromosomal inversion and translocation, 111.14 ± 53.24 pg/ml and 158.01 ± 62.34 pg/ml, respectively. Otherwise abnormalities included 47,XYY, chromosomal deletion and other karyotypes. And mean serum inhibin B was 96.43 ± 46.31 pg/ml.

### Y chromosome microdeletions and inhibin B

As shown in Table 5, fifty-eight (18.0%) of 322 NOA patients had microdeletions in the AZF region. Among these patients with YCMD, the most common site of YCMD in 20 of them was AZF c + d, with an incidence of 6.5%, and inhibin B concentration was 89.98 ± 50.11 pg/ml. AZF c and AZF d site YCMD had pretty much the same level of inhibin B levels, which were 93.37 ± 61.82 pg/ml and 82.17 ± 64.55 pg/ml, respectively.

**Table 5:** Y chromosome microdeletions and inhibin B

		No		%		Inhibin B (pg/ml)
No deletion		264		82.0		115.04 ± 89.30
Non-AZF a&b		35		10.9		86.34 ± 69.65
	AZF c		12		3.7	93.37 ± 61.82
	AZF d		3		0.9	82.17 ± 64.55
	AZF c + d		20		6.2	89.98 ± 50.11
AZF a&b		23		7.1		38.80 ± 27.14
	AZF a		2		0.6	16.05 ± 1.53
	AZF b		6		1.9	56.16 ± 39.54
	AZF b + c		5		1.6	19.86 ± 24.05
	AZF b + c + d		2		0.6	28.89 ± 25.46
	AZF a + b + c + d		8		2.5	10.14 ± 15.22
Total		322		100.0		84.01 ± 63.17
				*P* Value		<0.05 (1-way ANOVA)

**Fig. 3:**
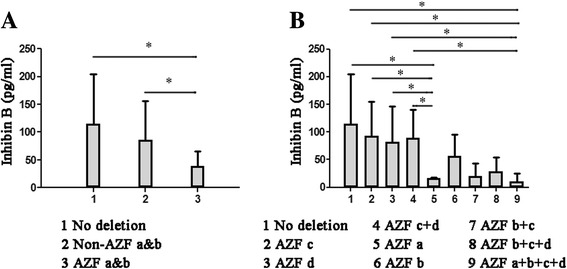
Serum inhibin B levels in (**a**) men with AZF a&b site Y chromosome microdeletions patients (YCMD), Non-AZF a&b site YCMD and no deletion and (**b**) different YCMD

Compared with Non-AZF a&b site YCMD deletion which contained AZF c + d, AZF c and AZF d, inhibin B was low in AZF a&b site YCMD deletion which contained AZF a, AZF b, AZF b + c, AZF b + c + d and AZF a + b + c + d. Compared to men with AZF a&b site YCMD deletion, those with Non-AZF a&b site YCMD deletion and no deletion had increased inhibin B on univariate analysis (Fig. 3a, p<0.05). Among twenty-three patients with AZF a&b site YCMD, the most frequent site in 8 of them was AZF a + b + c + d, with an incidence of 2.5%, and inhibin B concentration was 10.14 ± 15.22 pg/ml. Followed by AZF b, AZF b + c, AZF a and AZF b + c + d, inhibin B concentrations were 56.16 ± 39.54 pg/ml, 19.86 ± 24.05 pg/ml, 16.05 ± 1.53 pg/ml and 28.89 ± 25.46 pg/ml. Compared to men with other subtypes, those with AZF a or AZF a + b + c + d site YCMD deletion had decreased inhibin B on univariate analysis (Fig. 3b, p<0.05).

## Discussion

Infertility became a relatively common problem which affected couples in the world wide. For the most part of infertility remained idiopathic, and more and more genetic factors were demonstrated to affect fertility. As the main objective of this study was to discuss the relationship of genetic causes and inhibin B in NOA spermatogenic failure, we focused here on NOA classified according to histopathological findings and obstructive azoospermia was exclusive.

Testicular biopsy was routinely performed for diagnostic purposes in azoospermic patients. However, parts of NOA did not perform biopsy as the volume of testis only 1–4 ml. These patients were afraid of failure in TESA or TESE operation and chose donor semen. Relying on micro-TESE, successful sperm retrieval can be achieved in azoospermic patients with very small testicles, such as Klinefelter syndrome. It should be better to encourage these patients to try to find their own sperm in future. In addition, histopathological findings in testicular biopsy specimens did not show histopathology of the entire testicle. The absence of spermatozoa in one testicular biopsy did not preclude the presence of spermatozoa in another part of the testis. In order to minimize the wound, only one site was chosen in testicular biopsy procedure. To identify focal spermatogenesis, multiple biopsies are usually obtained at TESE.

The Sertoli cells play a key role in regulation of spermatogenesis and signaling in the testis by serving as the targets for FSH and testosterone. Sertoli cell functions include production of a number of proteins which regulate and respond to pituitary hormone release, and transducing those endocrine signals and other cellular cues into paracrine regulation of germ cells [10, 11]. FSH has considerable biological role in testicular function. Lacking of FSH would shorten testis size, sperm concentration and sperm motility, so FSH plays a important role in boosting cells proliferation and is desired for normal sperm production [12]. FSH induces the Sertoli cells to secrete inhibin and androgen-binding protein and plays a major role in initiation and progression of spermatogenesis. Inhibin acts as an FSH inhibitor secreted from the Sertoli cells, while activin is secreted by the Sertoli cells and the pituitary gland and stimulates FSH secretion [13]. Inhibin B, which is likely to be the crucial feedback regulator of FSH secretion, is associated well with FSH in sperm concentration, and thus support them as serum characters of spermatogenesis [7]. While inhibin B is maintained in detectable serum levels, spermatogenic activity in adults is required [14]. The inhibin B levels were dramaticlly lower in male with a spermatogenic defect. In adult male, inhibin B concentration is set and can be considered to be an index of Sertoli cell number and integrity. In our study, we found that inhibin B tended to be opposite to FSH in the same group. Therefore, FSH and inhibin B concentration conjointly reflected the capacity of spermatogenesis in MA was better than SCO and non-biopsy but worse than hypospermatogenesis. The understanding in advantage of inhibin B measurement is enhanced gradually. Inhibin B has been reported to be a more sensitive factor in study of azoospermic men than the testis size, FSH, and even the testis biopsy [15]. Inhibin B reflects the function of the total testicular tissue, whereas biopsies are not representative of the entire testis.

The genetic basis of NOA is unknown in the majority of infertile men [16]. So far, many studies have been done on not just infertile men with chromosomal abnormalities, but also mutations in genes that have been associated with NOA. For example, TEX11, testis expressed gene 11, may play critical role in meiotic recombination, genome integrity, and gametogenesis [17, 18]. And TEX15, testis expressed gene 15, is required for meiotic recombination and chromosomal synapsis in males [19].

Genetic testing is recommended when ICSI is performed or sperm concentration < 5 × 10^6^ /ml [2]. The correlation between a higher frequency of genetic abnormality and the severity of the testicular phenotype became a consensus gradually, and genetic examine became a part of the procedure selected in diagnostic workup. Like it has been reported a strong association between AZF microdeletions and chromosomal abnormalities, it was found that there was a high correlation between karyotypes and YCMD in current study. As a high correlation between karyotypes and YCMD, relationship of genetic causes and inhibin B was considered in two parts separately.

Chromosomal aneuploidy involves a change in chromosomal number from the standard diploid chromosomal complement. Most common of chromosomal aneuploidy in NOA were 47, XXY, named Klinefelter syndrome (KS). The incidence of KS accounted for about 10.6% in azoospermic male in this study, which was similar with the range of published studies [20, 21]. Inhibin B concentration in KS was merely 10.49 ± 3.81 pg/ml, significant lower than most other types of chromosomal abnormality. The karyotype with the lowest level of inhibin B in this study was 46,XX or 45,X. The level of inhibin B in current study conformed to the theory that presence of an extra non-synapsed Y chromosome activated checkpoint mechanisms to prevent the progression of meiosis, leading to dyszoospermia [22], a superabundance of X chromosomes could affect spermatogenesis [23], and loss of a chromosome should be more harmful than more of a chromosome [24].

Mosaic aneuploidy, which deeply affects the genetics, may be a novel mechanism for generating phenotypic diversity driven by genomic plasticity [25]. A mosaic karyotype is often accompanied by a rare sexual development disorder. Adults may vary in degree of infertility depending on the amount of mosaicism. Since many studies on infertile men with mosaic aneuploidy have shown that the majority of germ cells in meiosis are of normal karyotype, and the cells with abnormal karyotypes are progressively eliminated [26]. And mosaics of 46,XY/47,XXY, 46,XY/45,X and other karyotypes were detected higher inhibin B concentration. So spermatogenesis of mosaic karyotypes was better than pure 47,XXY, 46,XX and 45,X, depending on the ratio of 46,XY dose effect [27].

Chromosome polymorphisms were detected lower inhibin B concentration than normal karyotype in this study, such result were in accordance with previous reports that chromosome polymorphisms disorders mainly harmfully affect sperm concentration [28]. Surprisingly, the level of inhibin B in chromosome inversion and translocation disorders was higher than normal karyotype. It may be associated with the difference of patient histopathological characteristics. The azoospermic males with SCO were contained in the normal karyotype part, so their level of inhibin B was relatively low. Spermatogenic arrest in translocation carriers was due to a structural effect related to complex meiotic configurations. Chromosome 1 harbored a critical domain for genes important in azoospermia [29]. These chromosome inversion and translocation orders could cause developmental early pregnancy loss, habitual miscarriage and even birth defects. Inversion loop could lead to a breakdown in meiosis and an apoptosis in cells [30], and translocated chromosomes would also disturb and impede meiosis, resulting in varying extents of spermatogenic impairment [31]. Early study showed that the cases of live birth for balanced translocations, inv.(9), Robertsonian translocation, and inversions were 85.7%, 100%, 83.3%, and 75% respectively [32]. And inhibin B concentration in current study suggested that these NOA patients may still have residual spermatogenesis. Men who were proven fertile did not routinely perform hormone analysis in our center. But it would be better to have a proper control in this study using fertile men. We should gain information to see whether the levels of inhibin B in the NOA men are significantly lower compared to fertile men as a control.

At present, there are four different spermatogenetic loci azoospermia factors (AZFa, AZFb, AZFc and AZFd) have been detected in the long arm of the Y chromosome. Deletions within the AZF region in NOA existed 18.0% in the study, similar with other published studies [33]. Compared to no deletion, inhibin B concentration in YCMD was much lower. These findings were in accordance with that microdeletion in different AZF region could result in different degrees of spermatogenic failure [34]. Candidate genes inside the AZF regions have been researched extensively and are considered to play critical roles in regulation to germ cell cycle and meiosis, but it has not get the consensus of the molecular basis for deficient spermatogenesis [35].

On the basis of inhibin B concentration, YCMD was classified to Non-AZF a&b which contained AZF c + d, AZF c and AZF d and AZF a&b which contained AZF a, AZF b, AZF b + c, AZF b + c + d and AZF a + b + c + d. Non-AZF a&b microdeletion were detected higher inhibin B concentration than AZF a&b microdeletion. Such results indicated that spermatogenesis within Non-AZF a&b region deletion was better than AZF a&b microdeletion, and were in accordance with previous reports [36, 37]. The AZFa region deletion is often the most severe with individuals often presenting with SCO syndrome. Deletion within the AZFb region is associated with azoospermia, too; but it is an outcome of MA during the early meiotic stage. Deletions within the AZFc and AZFd regions are most associated with hypospermatogenesis which may result in cryptozoospermia or azoospermia. A complete AZFb region deletion predicts the absence of testicular spermatozoa. The AZFb + c microdeletion patients usually had a poor success rate of finding sperm for use in ICSI, and AZFc + d microdeletion patients generally could obtain sperm through the use of TESA or TESE.

There were some limitations that needed to be taken into account. Hypospermatogenesis, which was one kind of NOA, was better to classified to mild, moderate and severe. Considering the standard consensus of histopathological hypospermatogenesis was not built, this study did not perform detailed distinction. Although emphasis in study was inhibin B, it was better to evaluate the status of spermatogenesis combining FSH and inhibin B. Finally, we recommended performing Y chromosome microdeletion test in oligozoospermia and not only in azoospermia.

## Conclusions

In conclusion, there was a high correlation between karyotypes and YCMD. According to the level of inhibin B, spermatogenesis in chromosomal abnormality from lowest to highest was 46,XX (or 45,X), 47, XXY, mosaics, polymorphisms, inversion and translocation. And spermatogenesis within Non-AZF a&b region deletion was better than AZF a&b microdeletion.

## Abbreviations

FSH: Follicle stimulating hormone
ICSI: Intracytoplasmic sperm injection
MA: Maturation arrest
NOA: Non obstruction azoospermia
OA: Obstruction azoospermia
SCO: Sertoli cell only
